# Delayed shifts in salivary microbiota composition following long-term use of 0.06% chlorhexidine toothpaste

**DOI:** 10.3389/froh.2026.1887909

**Published:** 2026-08-11

**Authors:** Veronika Chuchmova, Kristýna Brodíková, Martina Zapletalova, Mark Lindholm, Martin Krsek

**Affiliations:** 1 Department of Public Health, Faculty of Medicine, Masaryk University, Brno, Czechia; 2 Department of Biochemistry, Faculty of Science, Masaryk University, Brno, Czechia; 3 Division of Oral Health and Periodontology, Department of Dental Medicine, Karolinska Institutet, Stockholm, Sweden

**Keywords:** antimicrobial resistance, chlorhexidine digluconate, oral bacteria, oral microbiota, prediction of resistance, salivary microbiota, antiseptics

## Abstract

**Background:**

Chlorhexidine is a standard antiseptic in periodontal therapy, yet its long-term impact on the commensal oral microbiota and antimicrobial resistance remains unclear.

**Objective:**

This study evaluated the ecological effects of 0.06% chlorhexidine toothpaste on the salivary microbiota of healthy individuals, focusing on taxonomic composition, functional shifts, and prevalence of predicted antimicrobial resistance pathways.

**Methods:**

This study was designed as a prospective, single-arm interventional trial to evaluate the temporal dynamics of the oral microbiota. Saliva samples were collected from eleven healthy participants at three specific time points: baseline (following a 4-week run-in period using a reference fluoride toothpaste), intervention (after 12 weeks of using 0.06% chlorhexidine digluconate toothpaste), and washout (after a subsequent 12-week period using the reference fluoride toothpaste). Sequencing targeted the 16S rRNA V3–V4 region.

**Results:**

Alpha diversity remained stable throughout the study. However, beta diversity analysis revealed significant shifts in community structure, which were most pronounced between the intervention and washout phases. We identified forty-one differentially abundant metabolic pathways, including a significant downregulation of predicted beta-lactam resistance pathways during the washout period. While chlorhexidine did not increase predicted resistance-related pathways, delayed compositional shifts toward gram-negative taxa were observed at the end of the 12-week washout period.

**Conclusion:**

These findings suggest that 0.06% chlorhexidine is not ecologically neutral, and dental professionals should carefully consider the appropriateness of chlorhexidine for long-term use.

**Clinical Trial Registration:** ClinicalTrials.gov, NCT07570927.

## Introduction

Oral health depends critically on the ecological balance of the oral microbiota. While a stable oral microbiota supports mucosal defense and homeostasis, its disruption or dysbiosis precipitates common oral diseases, including periodontitis and dental caries (1, 2). This ecosystem is significantly influenced by oral hygiene habits and chemical agents applied. Antiseptics are widely used in dental products to suppress oral pathogens; however, it is crucial to determine whether these agents act selectively or if they also indiscriminately reduce beneficial commensal bacteria (3, 4).

Chlorhexidine (CHX) is a broad-spectrum antiseptic widely used in clinical dentistry for its antimicrobial properties (5). Dental products containing CHX that are currently available to the public feature varying concentrations of this antiseptic and are marketed in diverse formulations, including mouthwashes, toothpastes, and gels (6). For routine or full-mouth applications, the highest concentrations in commercially available over-the-counter products reach approximately 0.2% CHX; formulations containing 0.12% CHX or above are recommended for short-term use, typically limited to a maximum of 28 days (with higher concentrations restricted even further). Conversely, lower concentrations, such as 0.06% CHX, are clinically indicated for extended periods of up to 6 months, or are marketed for long-term use without explicit time restrictions (6, 7).

CHX's effectiveness in reducing oral bacterial load has been well-documented (8, 9), but its long-term impact on the salivary microbiota and its potential implications for antibiotic resistance remain areas of active research (5, 6, 10).

CHX exerts its antimicrobial activity by disrupting bacterial cell membranes. It generally displays higher effectiveness against gram-positive bacteria due to their higher negative surface charge, which facilitates strong binding with the cationic antiseptic. In contrast, gram-negative bacteria are less sensitive due to the complexity of their cell envelope, which generally impedes the rapid penetration of the antiseptic (11). This differential sensitivity suggests a potential for inducing ecological dysbiosis by altering the proportional representation of species. Long-term use of CHX could potentially lead to undesirable ecological changes that favor resistant strains or disrupt the diversity of commensal species (12, 13). Understanding these changes is essential for assessing the broader impacts of CHX use on oral health.

Antimicrobial resistance (AMR) represents a significant global health challenge, with implications that extend beyond individual treatments (14). Consequently, clarifying the relationship between CHX use and AMR is of paramount importance for the dental community. Recent evidence suggests a possible link between exposure to CHX and the development of AMR. Specifically, *in vitro* studies have indicated reduced CHX sensitivity in bacterial strains that already exhibit resistance to certain antibiotics (15, 16). Furthermore, emerging evidence suggests that CHX exposure may drive the development of cross-resistance to clinically relevant antibiotics (17). However, it is important to note that these findings are predominantly based on *in vitro* models utilizing sub-lethal concentrations of CHX, which differ from those applied in clinical practice. To date, there is a lack of *in vivo* studies investigating this relationship in the context of commercial dental products and standard usage protocols. Therefore, investigating the effects of CHX on the emergence and persistence of antibiotic-resistant strains in the oral microbiota is crucial for guiding safe and effective clinical usage.

Therefore, the aim of this study was to investigate the longitudinal effects of chlorhexidine on the composition and functional potential of the salivary microbiota in healthy individuals. The study was designed to address the research question of whether daily use of 0.06% chlorhexidine digluconate induces ecological shifts in the salivary microbiota and alters the prevalence of predicted AMR markers.

## Material and methods

### Study and participants

This single-arm, prospective, longitudinal interventional study was conducted in strict adherence to the pre-established study protocol, which is publicly archived and accessible at https://dx.doi.org/10.17504/protocols.io.ewov1244ogr2/v1. To ensure maximum transparency, reproducibility, and methodological rigor, the study design and experimental workflow were structured in accordance with the STORMS (Strengthening the Reporting of Microbiome Studies (18), development framework, and followed the specialized clinical guidelines for oral microbiome research proposed by Zaura et al. (19).

The study was approved by the Ethics Committee of the Faculty of Medicine, Masaryk University (Approval No. 68/2020). All participants were fully informed about the nature, scope, and potential risks of the research, and provided written informed consent prior to their formal inclusion in the study cohort.

Participants were recruited from the local community in Brno, Czech Republic, between March and April 2022, through direct personal communication by the principal investigator. To ensure a final target sample size of eleven despite anticipated attrition, an initial cohort of 14 young, non-smoking, systemically healthy adults from a single geographical region was enrolled. Selection followed strict criteria detailed in the Study Protocol; briefly, eligible participants had to be unmedicated, with no antibiotic therapy in the preceding 6 months and no special dietary regulations.

Dental eligibility was confirmed via clinical examination. Inclusion required an intact natural dentition free of untreated caries, inflammatory periodontal diseases, dental implants, or orthodontic appliances. To minimize confounding effects of biofilm variability and ensure uniform antiseptic exposure, only participants with excellent baseline oral hygiene were selected [Plaque Index < 0.2; Gingival Index < 0.143; Silness and Löe (20)]. Regular use of interdental cleaning aids was required, and hygiene habits were monitored at every sampling point to ensure consistency. During the study, three participants were excluded: one due to relocation, one due to antibiotic treatment, and one who discontinued the intervention due to extrinsic tooth staining. Baseline characteristics are summarized in Table 1.

**Table 1:** Baseline and clinical characteristics of the study population.

Gender	Female:Male	6:5
Age	30.4 ± 5.8	Range: 25–43
PI	0.023 ± 0.06	Range: 0–0.2
GI	0.052 ± 0.04	Range: 0–0.14
Toothbrush type	Manual only	8 (72.7%)
	Manual + Sonic combined	3 (27.3%)
Interdental cleaning	Interdental brushes (IDB) only	8 (72.7%)
	IDB + Floss combined	3 (27.3%)

Data are presented as mean ± standard deviation (range) or as absolute frequency (percentage). PI, plaque index; GI, gingival index; IDB, interdental brush. Clinical indices were assessed according to the methodology described in the study protocol.

### Study design and sampling timeline

The trial comprised three consecutive phases, during which participants were restricted to the dental preparations supplied by the investigators; the use of other treatments like oral probiotics, natural rinses, or additional antiseptic products was prohibited.

During the initial 4-week run-in period (Phase 1; baseline), participants maintained their habitual mechanical oral hygiene practices, including manual or electric toothbrushes and interdental cleaning aids, but were required to use only a provided standard non-medicated toothpaste (Elmex® JUNIOR, 1,400 ppm amine fluoride) to establish a stable microbiological baseline. This was followed by a 12-week intervention period (Phase 2; CHX intervention), during which participants replaced the reference fluoride toothpaste with a test toothpaste containing 0.06% chlorhexidine digluconate, used twice daily according to their established hygiene routine. Finally, during the 12-week washout period (Phase 3; washout), participants reverted to the original reference toothpaste to evaluate the reversibility of the expected microbial shifts. The exact formulation and active ingredients of both dentifrices are detailed in Table 2. Throughout all phases, participants were required to report any antibiotic treatment, which resulted in immediate exclusion from the study. Furthermore, clinical health status and dental indices were monitored at every sampling point to ensure that oral health and hygiene performance remained consistent throughout the entire 28-week duration.

**Table 2:** Summary of the toothpaste types used in the study.

Toothpaste type	Complete ingredient list
Reference (baseline & washout)	Aqua, Hydrated Silica, Sorbitol, Hydroxyethylcellulose, Olaflur 1,400 ppm, Aroma, Saccharin, Limonene. * Commercially available as Elmex JUNIOR, CP GABA, Świdnica, Poland
Intervention (0.06% CHX)	Aqua, Sorbitol, Hydrated Silica, Isomalt, Panthenol, Hydroxyethylcellulose, PEG-40 Hydrogenated Castor Oil, Cocamidopropyl Betaine, Aroma, Titanium Dioxide, Sodium Fluoride 1,450 ppm, Sodium Saccharin, Sodium Chloride, Aloe Barbadensis Leaf Juice, Sodium Methylparaben, Glycerin, Chlorhexidine Digluconate 0,06%, Cetylpyridinium Chloride, Tocopherol, Sodium Benzoate, Limonene. * Commercially available as GUM Paroex, Sunstar, Barcelona, Spain

### Sample collection, DNA extraction, and sequencing

Unstimulated whole saliva samples were collected at the end of each study phase into sterile tubes. To minimize diurnal variability, sampling was consistently performed in the morning before brushing teeth and consuming any food or drink. Participants provided the samples via active expectoration until a standardized volume of 5 mL was reached, with the entire collection process taking no longer than 9 min. Samples were immediately frozen at −20 °C until further processing.

Total bacterial DNA was extracted using the DNeasy® PowerSoil® Pro Kit (Qiagen, Hilden, Germany) according to the manufacturer's instructions. The V3–V4 hypervariable regions of the 16S ribosomal RNA (16S rRNA) gene were amplified using the standard Illumina 16S metagenomic sequencing library preparation protocol. The specific primers used were: Forward 5′-CCTACGGGNGGCWGCAG-3′ and Reverse 5′-GACTACHVGGGTATCTAATCC-3′ (21). Paired-end sequencing (2 × 300 bp) was performed on the Illumina MiSeq platform (Illumina Inc., San Diego, CA, USA) at BioVendor—Laboratorní medicína a.s. (Brno, Czech Republic).

### Bioinformatic processing and statistical analysis

Demultiplexed raw reads underwent quality control, adapter trimming, denoising, and consensus chimera removal using the DADA2 pipeline (implemented within QIIME 2) to generate Amplicon Sequence Variants (ASVs) (22). Taxonomic assignment was performed using a Naive Bayes classifier trained against the SILVA reference database (version 138.1) with a confidence threshold of 0.7. To remove rare, low-frequency, and potentially spurious features, ASVs were filtered during the bioinformatic pipeline to retain only robust, annotated features for downstream analysis.

Downstream statistical evaluation and data visualization were performed using MicrobiomeAnalyst 3.0 with further data filtering disabled to preserve the pre-filtered ASV structure (23). Alpha diversity (Shannon and Simpson indices) was compared using Mann–Whitney *U* or Kruskal–Wallis tests, as appropriate. Beta diversity was visualized via Principal Coordinates Analysis (PCoA) and calculated using two complementary metrics: Aitchison distance [Euclidean distance on Centered Log-Ratio (CLR) normalized data] and Bray–Curtis dissimilarity on Total Sum Scaling (TSS) normalized data. Differences in overall microbial community composition between timepoints were formally tested via Permutational Multivariate Analysis of Variance (PERMANOVA) with 999 permutations, followed by *post-hoc* pairwise comparisons. Functional prediction of the salivary microbiota, including metabolic potential and AMR pathways, was conducted using PICRUSt2 (Phylogenetic Investigation of Communities by Reconstruction of Unobserved States 2) (24).

Differential abundance analysis was performed at the phylum, family, genus, and species levels to identify shifts between the baseline, intervention, and washout phases. DESeq2 was employed as the primary statistical tool. To ensure robustness, a sensitivity analysis was conducted using edgeR (Empirical analysis of digital gene expression data in R) with identical threshold settings, and concordance between the two methods was evaluated. Taxa showing nominal significance (*p* < 0.05) were also recorded to identify potentially biologically relevant features warranting further investigation.

Multiple testing corrections were performed using the Benjamini–Hochberg method to control the false discovery rate (FDR). For functional pathways predicted by PICRUSt2, significance was assessed using adjusted *p*-values (*p*-adj). In both cases, a threshold of <0.05 was considered statistically significant.

Generative artificial intelligence tools were utilized in the preparation of this manuscript for visual and linguistic refinement. Specifically, Figure 6 (*Longitudinal dynamics of predicted antimicrobial resistance pathways*) was generated and visually formatted using ChatGPT (GPT-4 architecture, OpenAI, San Francisco, CA, USA) based on analyzed data from PICRUSt2. Additionally, language refinement of the manuscript text was assisted by the Gemini large language model (Google LLC, Mountain View, CA, USA).

## Results

A total of 33 sequenced samples from 11 participants were analyzed. To ensure consistent oral health status, dental indices were determined at each of the three sampling time points (baseline, post-intervention, and post-washout). Throughout all three phases of the project, no significant changes in dental index values were recorded, adhering to the inclusion criteria defined in the Study Protocol. All salivary samples yielded high-quality raw reads ranging from 61,000 to 142,000 reads per sample. Following bioinformatic processing and quality filtering through the DADA2 pipeline, a total of 233 ASVs were identified.

Taxonomic classification using the SILVA reference database (v138.1) identified 8 bacterial phyla, 14 classes, 33 orders, 47 families, 87 genera, and 117 species.

### Microbial diversity

**Figure 1:**
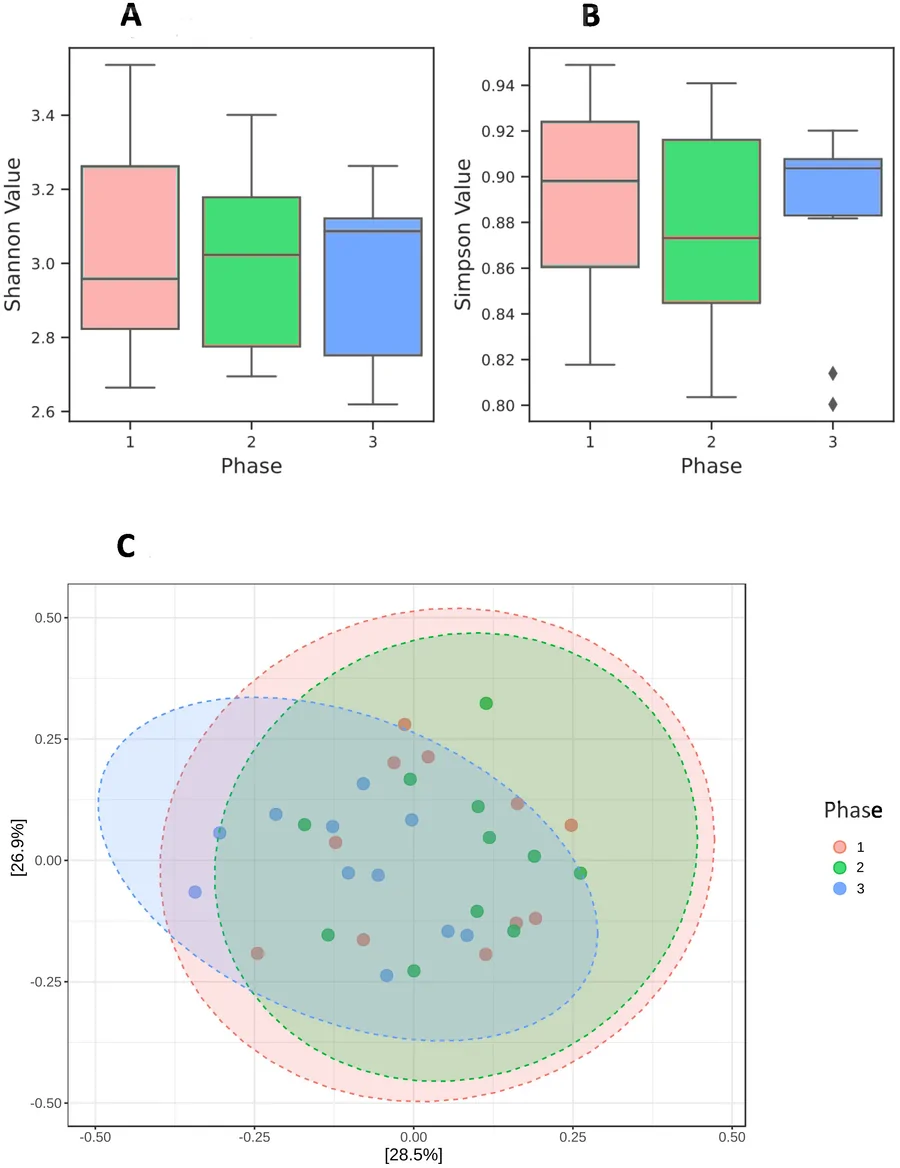
Quantitative analysis of community profiles. Shannon's index **(A)** and Simpson index **(B)** representing alpha-diversity with no statistical significance (*p* > 0.05) between phases 1 (baseline), 2 (CHX intervention), and 3 (washout). Principal Coordinate Analysis (PCoA) plot based on Bray–Curtis dissimilarity representing beta-diversity **(C)**, showing a significant shift in community structure between phases (*p* < 0.05).

To assess the impact of chlorhexidine intervention on salivary microbiota ecology, alpha and beta diversity across the study phases were evaluated. No statistically significant differences in species richness or evenness were detected between phases via alpha diversity analysis using Shannon and Simpson indices (*p* > 0.05; Figures 1A,B). In contrast, beta diversity analysis revealed a statistically significant shift in salivary microbial community structure across the study phases, consistent across both evaluated metrics (PERMANOVA, *p* < 0.05). Specifically, overall community differences were significant when assessed by both Bray–Curtis dissimilarity (*p* = 0.03, Figure 1C) and Aitchison distance (*p* = 0.022). Pairwise comparisons confirmed that this temporal shift was driven specifically by a significant difference between Phase 2 and Phase 3 (Bray–Curtis, FDR = 0.018; Aitchison, FDR = 0.006).

### Taxonomic composition and differential abundance

To identify the drivers of the observed community shifts, we further analyzed changes in bacterial composition at the taxonomic levels.

**Figure 2:**
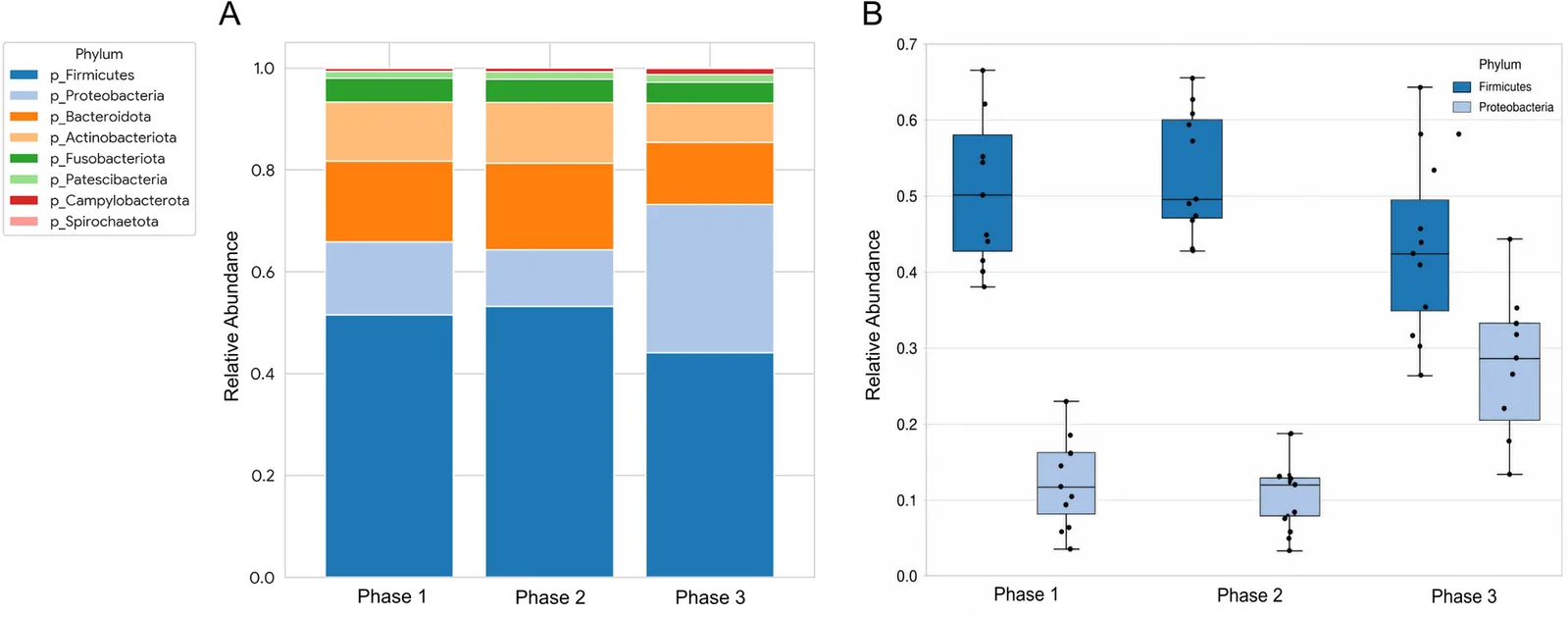
Temporal dynamics of salivary bacterial phyla across study phases. **(A)** Aggregate community composition. Stacked bar plot representing the mean relative abundance of bacterial phyla at baseline (Phase 1), after CHX intervention (Phase 2), and after the washout period (Phase 3). **(B)** Phylum-level variations. Box plots showing the relative abundance of the two dominant phyla: Firmicutes (FDR > 0.05) and Proteobacteria (FDR < 0.05).

Taxonomic profiling at the phylum level identified Firmicutes as the predominant phylum across all study phases (Figure 2A). However, significant compositional shifts were detected, particularly at the end of the washout period. Compared to baseline, the relative abundances of Proteobacteria and Campylobacterota significantly increased (FDR < 0.05), whereas Actinobacteriota significantly decreased (FDR < 0.05). Furthermore, comparisons between the intervention and washout phases confirmed the significant expansion of Proteobacteria and Campylobacterota (FDR < 0.05). A reduction in Actinobacteriota and Bacteroidota was also observed in the washout period relative to the intervention period; however, these changes were not significant after correction for multiple testing (FDR > 0.05).

At the family level, the relative abundances of Pasteurellaceae and Campylobacteraceae were significantly higher in the washout period compared to baseline (FDR < 0.05). Significant increases in these families were also observed in washout period compared to the intervention period (FDR < 0.05).

**Figure 3:**
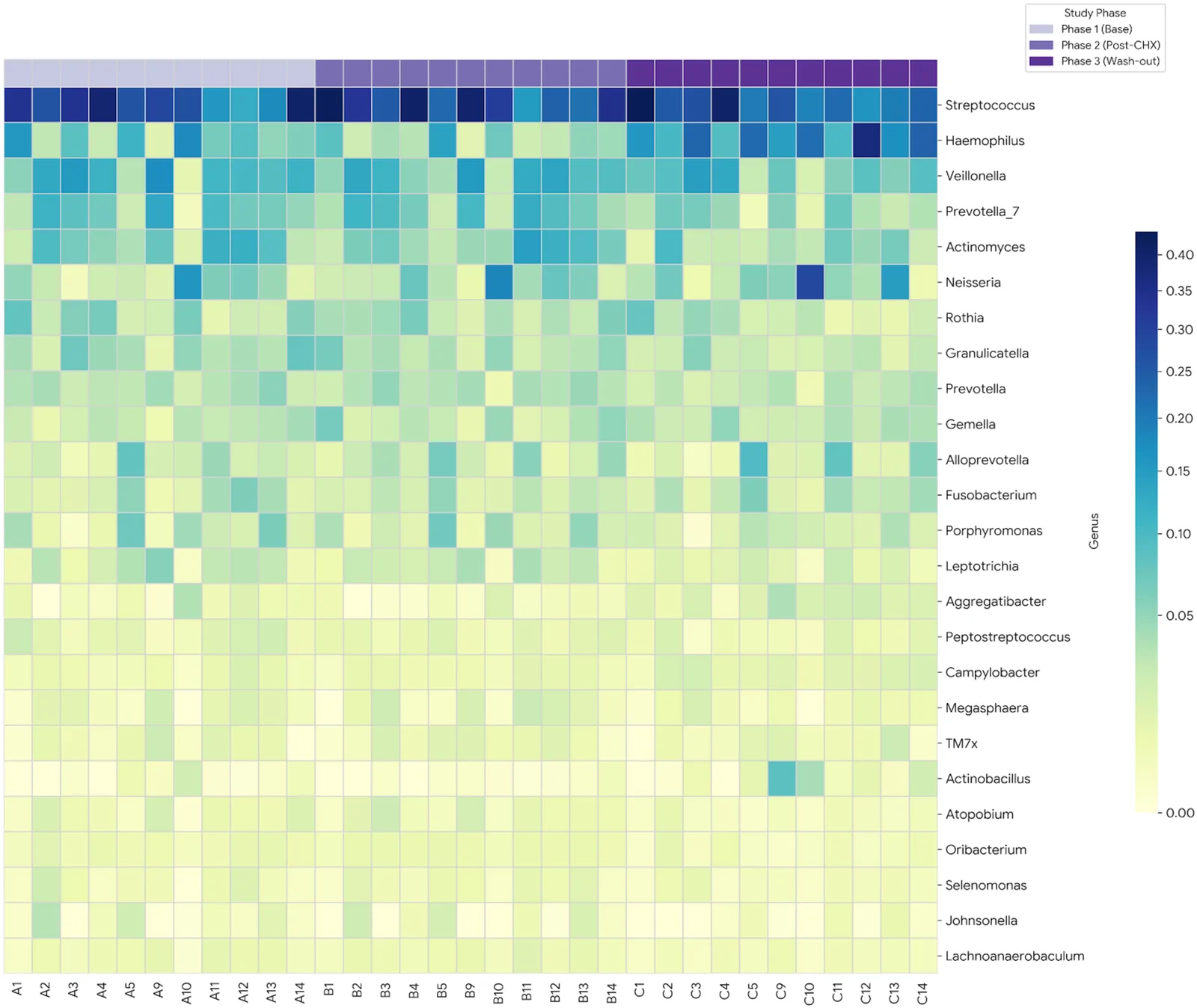
Heatmap analysis of taxonomic composition and relative abundance of the most prevalent oral genera across study phases. Heatmap visualizing the relative abundance of the 25 most prevalent genera across all study participants, sorted by study phase (Phase 1: Baseline; Phase 2: CHX intervention; Phase 3: Wash-out). Darker hues indicate higher relative abundance. To accommodate the high dynamic range of the data, color intensity is scaled using a power normalization (gamma = 0.5). This non-linear scaling enhances the visibility of low-abundance taxa (0%–15%) without oversaturating the dominant genus (*Streptococcus*).

Taxonomic profiles displaying bacterial genera and species across all study time points are provided in Supplementary Figures 1 and 2. To provide a comprehensive overview, the 25 most abundant genera were visualized in a heatmap (Figure 3). Consistent with these analyses, the salivary microbiota was dominated by *Streptococcus*, followed by *Haemophilus*, *Veillonella*, *Prevotella* (including the *Prevotella 7* group), and *Actinomyces*. Detailed analysis revealed that the predominant fraction of streptococci (mean 27.1% of total abundance) consisted of sequences unassigned at the species level. Phylogenetically, this cluster corresponds largely to the *Mitis* group (*Streptococcus mitis*, *Streptococcus oralis*) and exhibited high stability throughout the study, with no statistically significant fluctuations between phases. Other health-associated species (*Streptococcus parasanguinis*, *Streptococcus salivarius*) were detected at substantially lower abundances (<1%), while the cariogenic species *Streptococcus mutans* was present only in negligible quantities (<0.04%).

**Figure 4:**
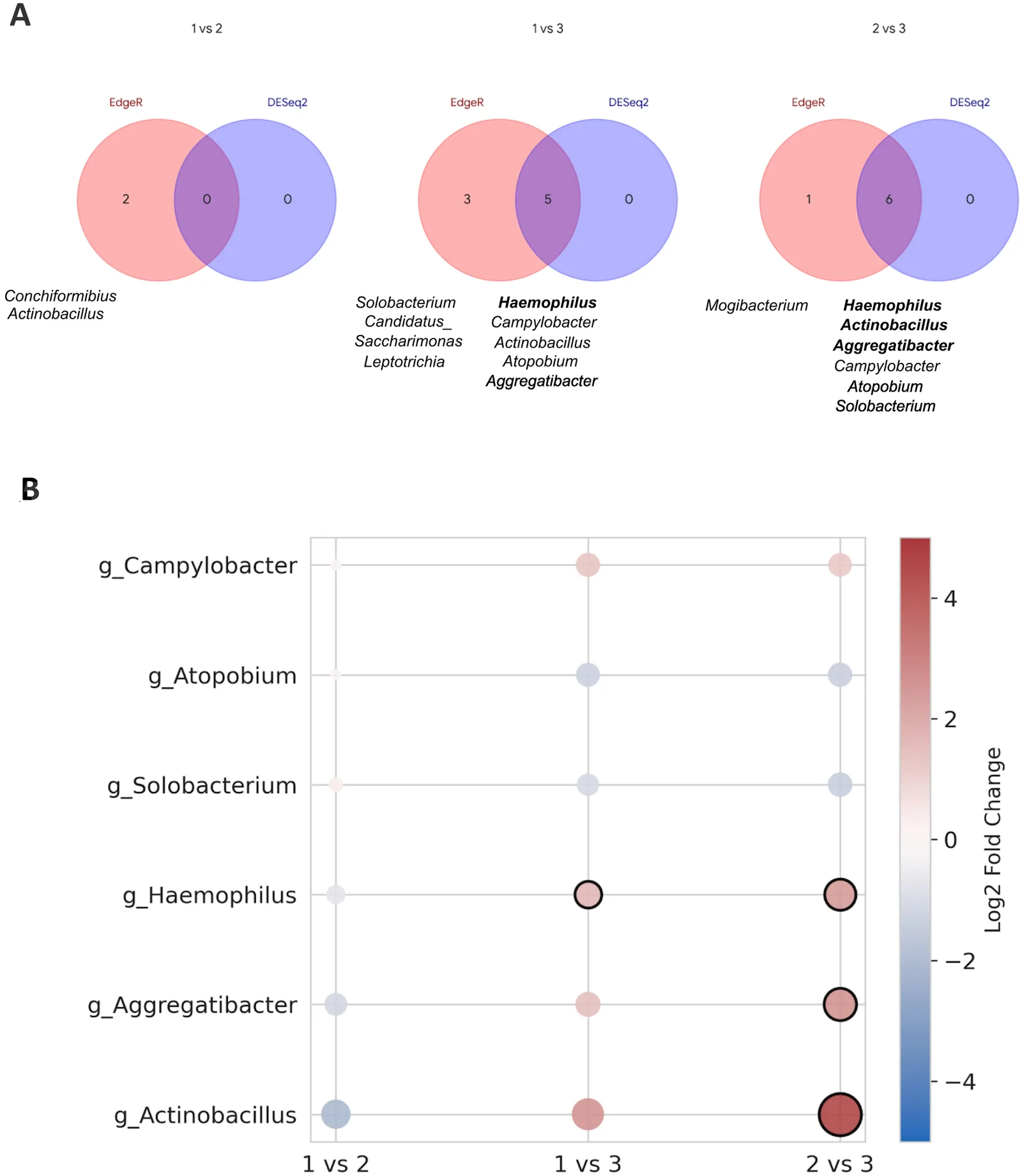
Temporal dynamics of salivary microbiota identified by a multi-method approach. **(A)** Methodological overlap and taxonomic identification. Venn diagrams illustrating the overlap of significant genera (*p* < 0.05) detected by EdgeR (red) and DESeq2 (blue) across the three comparisons. Associated bacterial genera are listed below each diagram, with those maintaining a False Discovery Rate (FDR < 0.05) highlighted in bold. **(B)** Differential abundance profiles. The bubble plot displays bacterial genera identified as significantly differentially abundant by the intersection of EdgeR and DESeq2 methods. The *X*-axis represents pairwise comparisons between study phases. The color gradient and bubble size correspond to the mean Log2 Fold Change; red bubbles indicate an increase, while blue bubbles indicate a decrease in the latter phase relative to the former. Bubbles with a black outline denote high statistical significance (FDR < 0.05 in both analyses).

To ensure robustness, differentially represented taxa were identified using the intersection of two statistical models (EdgeR and DESeq2), as illustrated in Figure 4A. This consensus approach confirmed that the shift in washout phase was primarily driven by the significant enrichment of *Haemophilus*, *Aggregatibacter*, *Actinobacillus*, and *Campylobacter* (FDR < 0.05). In contrast, *Solobacterium* and *Atopobium* were identified as taxa with significantly reduced abundance compared to baseline (Figure 4B).

At the species level, unassigned *Haemophilus* spp. were significantly more abundant in the washout phase compared to both baseline and the intervention phase (FDR < 0.05). Additionally, a significant increase in unassigned *Actinobacillus* spp. was observed in washout period when compared to intervention period (FDR < 0.05).

### Changes in salivary microbiota function and prediction of AMR potential

**Figure 5:**
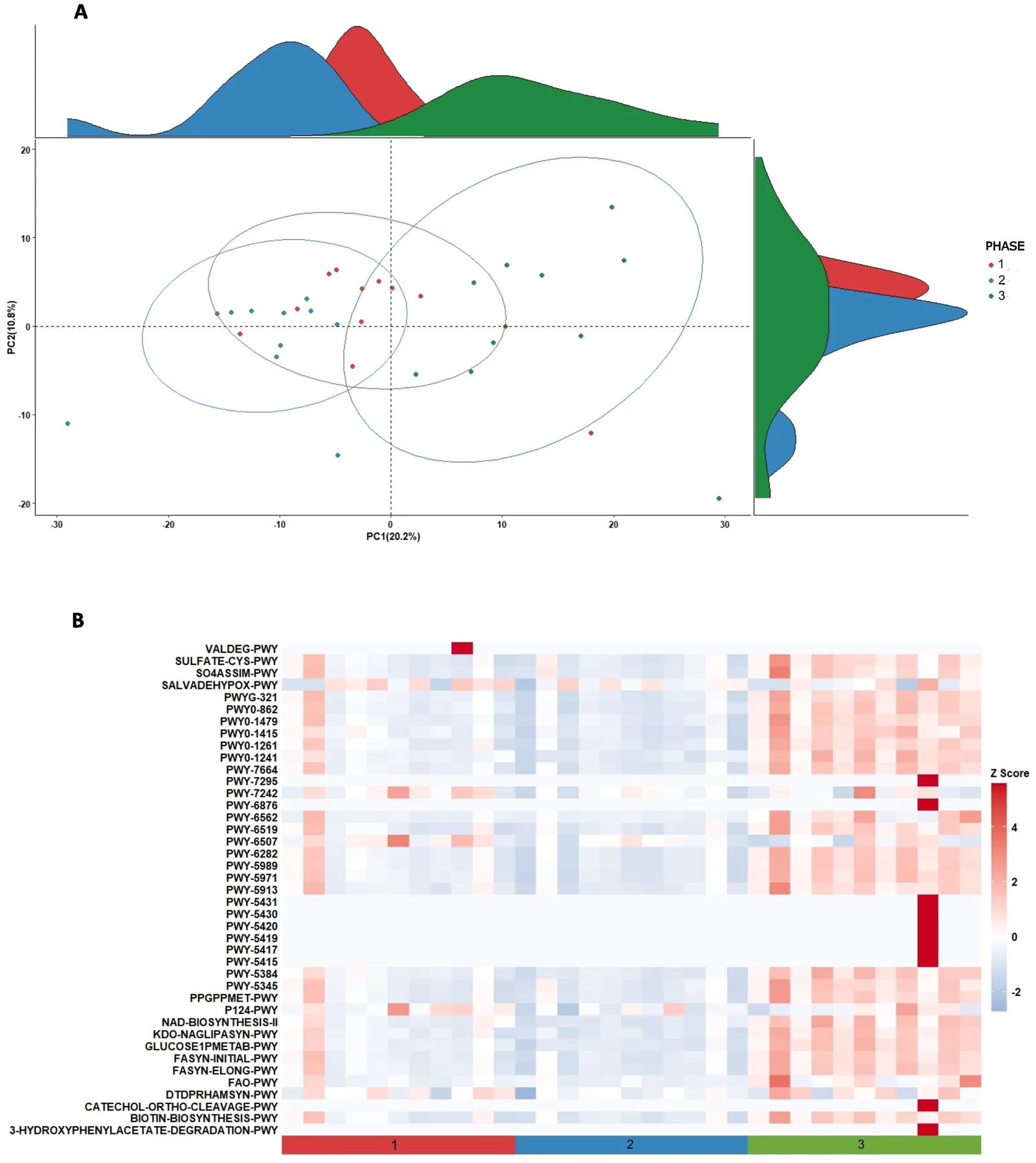
Predicted functional pathways of the salivary microbiota. **(A)** Principal Component Analysis (PCA) of predicted metabolic pathways showing distinct clustering of microbial communities across study phases: Phase 1 (baseline), Phase 2 (intervention), and Phase 3 (washout). **(B)** Heatmap of significantly altered metabolic pathways. The heatmap displays pathways with a *p*–adj < 0.05 between the intervention and washout phases.

Functional prediction analysis using PICRUSt2 and LinDA (Linear models for differential abundance analysis) identified distinct clustering of microbial communities across study phases (Figure 5A), indicating a significant overall shift in metabolic potential. Specifically, a total of 41 metabolic pathways with differing abundances between the intervention and washout phases were identified (*p*-adj < 0.05). These differentially abundant pathways are visualized in the heatmap (Figure 5B), while a subset of those with the highest statistical significance (*p*-adj < 0.01) is further detailed in Supplementary Figure 3.

The enriched pathways primarily represented functional categories related to the biosynthesis of cellular membrane components and essential cofactors. Specifically, pathways related to the superpathway of fatty acid biosynthesis initiation (FASYN-INITIAL-PWY), fatty acid elongation—saturated (FASYN-ELONG-PWY), biotin biosynthesis I (BIOTIN-BIOSYNTHESIS-PWY), and 8-amino-7-oxononanoate biosynthesis I (PWY-6519) were significantly enriched during the washout period compared to the intervention period (*p* < 0.01, *p*-adj < 0.04; Figure 5B). Similarly, pathways involved in lipopolysaccharide biosynthesis, such as the superpathway of (Kdo)_2-lipid A biosynthesis (KDO-NAGLIPASYN-PWY) and ADP-L-glycero-beta-D-manno-heptose biosynthesis (PWY0-1241) showed significant enrichment. In contrast, metabolic pathways involved in the nitrogen cycle (e.g., DENITRIFICATION-PWY, PWY-490-3) remained stable throughout the study period (*p* > 0.05).

**Figure 6:**
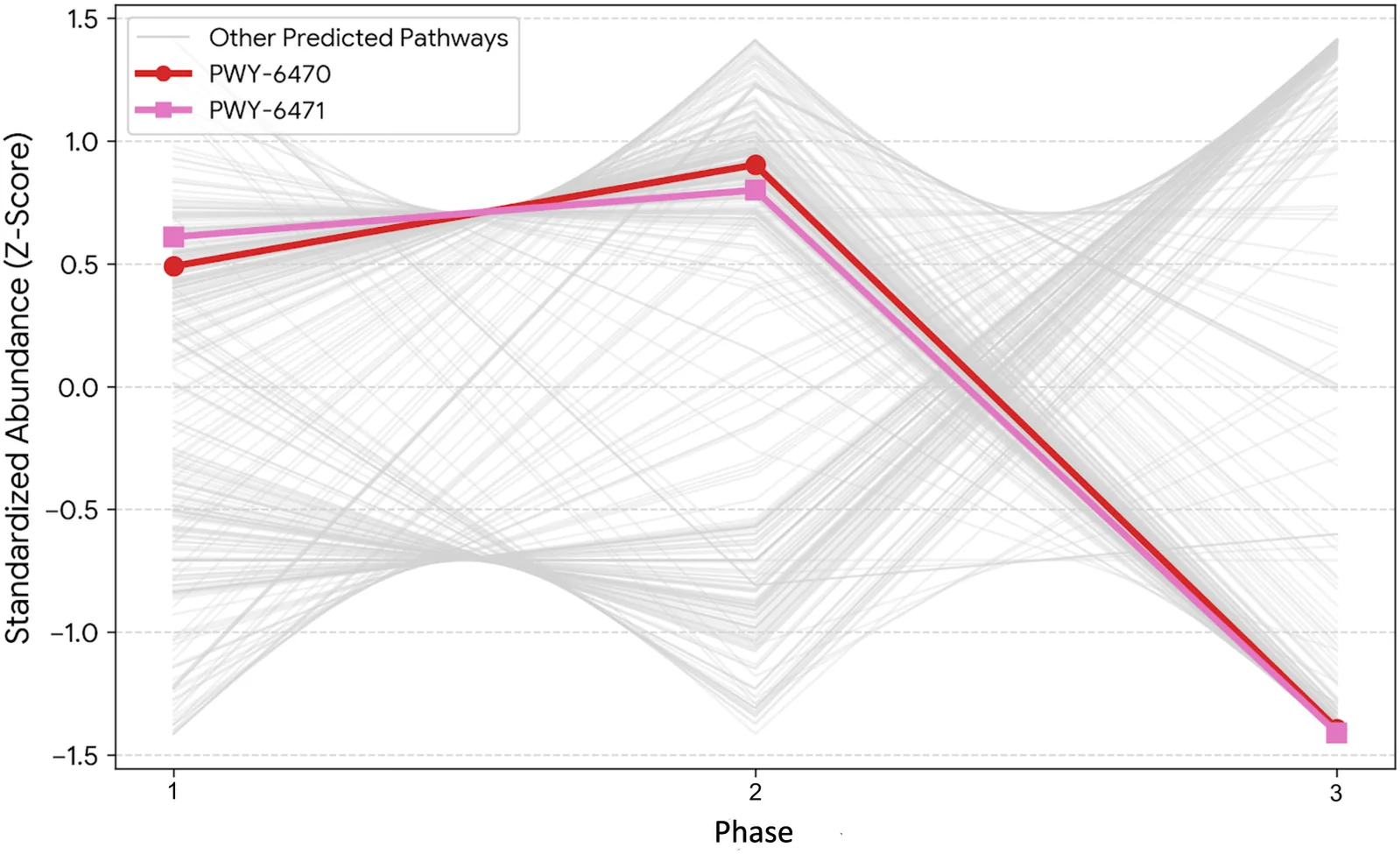
Longitudinal dynamics of predicted antimicrobial resistance pathways. Standardized abundance trends (*Z*-score) of all detected functional pathways across the study phases: Phase 1 (baseline), Phase 2 (intervention), and Phase 3 (washout). Grey lines illustrate the background community dynamics (*n* = 308 pathways), where the distinct upward trend observed during Phase 3 corresponds to the upregulation of pathways involved in the biosynthesis of cellular membrane components and essential cofactors, reflecting bacterial cell replication and community recovery. Colored lines highlight the specific trajectories of peptidoglycan biosynthesis pathways PWY-6470 and PWY-6471 (*p*-adj < 0.002).

Out of 310 predicted metabolic pathways, a targeted analysis was performed on pathways associated with antimicrobial resistance mechanisms. Statistical analysis revealed significant alterations in the abundance of peptidoglycan biosynthesis pathways linked to resistance. Specifically, pathways PWY-6470 and PWY-6471 associated with beta-lactam resistance were significantly downregulated in the washout phase compared to the chlorhexidine intervention phase (*p*-adj < 0.002, Supplementary Table 1). Longitudinal analysis of standardized abundance (*Z*-score) highlights the distinct dynamics of these markers against the global metabolic background. While the overall community profile showed variable trends, both identified resistance pathways followed a specific pattern of upregulation during the intervention, followed by a sharp decline after discontinuation (Figure 6).

## Discussion

Given the widespread long-term use of lower-concentration antiseptics, this study investigated the modulation of salivary microbiota by 0.06% chlorhexidine digluconate over a 12-week period and assessed the reversibility of these changes during a subsequent 12-week washout.

Since its introduction in the 1970s, the efficacy of chlorhexidine in reducing bacterial load and inhibiting dental plaque formation has been well established (7–9). However, this antiseptic also has associated drawbacks. While the most discussed side effect is tooth discoloration, which is well-documented particularly with long-term use of CHX mouthwashes (6, 7, 25), only one out of 14 participants in our cohort exhibited visible pigmentation after CHX toothpaste. Therefore, a biologically more significant concern is the alteration of the oral microbiota (4, 12).

### Taxonomic shifts and recolonization dynamics

In the present study, our analysis of the salivary microbiota revealed a significant shift from gram-positive to gram-negative taxa, which was primarily caused by a marked increase in Proteobacteria during the washout phase. Firmicutes remained the dominant phylum across all three phases of the study. However, the stable relative abundance of Firmicutes during the CHX intervention (Figure 2A) requires cautious interpretation. Given that CHX reduces the total bacterial load (5, 7), the observation that phylum proportions remained comparable to baseline implies a uniform suppression of the entire community. Consequently, while Figure 2B depicts a stable relative profile for Firmicutes in the intervention phase, comparable to baseline, absolute quantification would undoubtedly demonstrate a significant decline in Firmicutes abundance (and all other phyla). The ecological dynamics shifted only during the subsequent washout period, where we observed distinct compositional changes that moved away from the initial homeostatic state.

The impact of CHX on the oral microbiota as reported in current literature varies significantly, suggesting that the microbial response is influenced by both CHX concentration and study duration. Interestingly, our observation of a non-significant trend toward Proteobacteria reduction during the intervention phase aligns with similar findings by Wattanawongwan et al. (26), who also reported a decrease that did not reach statistical significance using a 0.12% CHX formulation. This shared observation of a downward trend at lower clinical concentrations stands in contrast to studies utilizing 0.2% CHX, where a significant and immediate enrichment of Proteobacteria was documented (12, 27). These differences suggest that while high doses of CHX can rapidly induce dysbiotic changes during the active phase of treatment, lower concentrations may have a more subtle suppressive effect on the salivary microbiota.

Interestingly, there were no statistically significant differences at the genus level between Phase 1 (baseline) and Phase 2 (CHX intervention) of our study. This finding contrasts with previous reports utilizing higher CHX concentrations (e.g., 0.12% or 0.2%), which have documented significant bacterial shifts after CHX treatment. However, consensus among these studies is lacking, with varying reports on which specific genera increase or decrease (12, 26, 28). In our study, changes in genus abundance were only apparent during the washout phase. The statistically significant genera enriched in Phase 3—*Haemophilus*, *Actinobacillus*, and *Aggregatibacter*—all belong to the phylum Proteobacteria, specifically the family Pasteurellaceae. Although the precise drivers of this synchronized surge remain to be fully elucidated, it is essential to interpret this increased abundance of gram-negative taxa with caution. Given that alpha diversity and clinical indices remained stable throughout our study, these microbial changes likely represent a compositional adaptation rather than a clinically relevant dysbiosis, particularly in individuals with a healthy oral microbiota.

To validate the composition of the baseline salivary microbiota, we compared our data with the core microbiome profile defined by Relvas et al. (29). We observed a high degree of concordance, with 14 of the 15 most abundant genera in our cohort matching the core taxa reported in their study. The only distinction was the presence of *Aggregatibacter* among the top 15 genera in our dataset (Supplementary Figure 1). Importantly, this minor discrepancy occurred despite both studies targeting the same 16S rRNA variable region (V3–V4) and utilizing the SILVA reference database, and it likely stems from database versioning. In earlier versions, sequences currently assigned to *Aggregatibacter* may have been categorized within the genera *Actinobacillus* or *Haemophilus*.

### Functional metabolic shifts, predicted resistance-related pathways, and systemic implications

Previous studies indicate that CHX impairs the ability of oral bacteria to reduce nitrates to nitrites (30), based on short-term (7 days) exposures to higher-concentration (0.2% CHX) mouthwashes (27, 31). In contrast, our results suggest that toothpaste with a lower concentration (0.06% CHX) does not affect this ability to reduce nitrates.

Significant shifts in metabolic pathways were most prominent during the washout phase, characterized by an enrichment of pathways associated with lipopolysaccharide (LPS) and cell envelope biosynthesis. Unlike peptidoglycan biosynthesis, which is common to most bacteria, LPS production is a hallmark of gram-negative taxa (32). Therefore, these functional increases directly align with the expanded abundance of gram-negative Proteobacteria observed during this period, providing robust functional evidence for the selective proliferation of this group.

Currently, there is a lack of studies investigating the impact of chlorhexidine on predicted AMR pathways *in vivo.* The predicted abundance of specific peptidoglycan biosynthesis pathways (PWY-6470 and PWY-6471) exhibited a minor, non-significant increase after CHX treatment, followed by a substantial and significant decrease during the washout phase. These dynamics correspond with the significant fluctuations observed in the abundance of the family Atopobiaceae throughout the study phases (*p* < 0.01). To the best of our knowledge, the only study examining this topic is the work by Bartsch et al. (33), who investigated the oral microbiota in patients exposed to 0.2% CHX. They examined both dental plaque and saliva and, with regard to AMR, focused specifically on the presence of resistance genes. Although they observed a non-significant increase in a tetracycline resistance gene following the CHX intervention, they did not find any statistically significant changes in the prevalence of AMR genes after FDR correction in either dental plaque or saliva, either after CHX treatment (4 weeks) or during the subsequent washout period (4 weeks).

The delayed shift toward gram-negative bacteria observed during the washout phase is noteworthy not only because of its importance for local oral health but also in the broader context of the oral-gut axis. Since the oral cavity serves as a continuous reservoir for microbes capable of translocating to the gastrointestinal tract (34), even these temporary compositional changes might have broader implications for systemic microbial homeostasis.

### Methodological strengths, limitations, and clinical relevance

The advantage of our study lies in the long-term monitoring of the salivary microbiota exposed to CHX and especially wash out, which provides a unique insight into the state of the salivary microbiota after CHX exposure. The selection of participants was also adapted to this monitoring. The selection of a group with excellent oral hygiene was a strategic decision to minimize microbial shifts associated with mature dental biofilm and gingivitis, which typically involve an increase in gram-negative and anaerobic bacteria (35, 36). Maintaining a consistent level of oral hygiene and dental plaque control throughout the entire 28 weeks was therefore crucial for this study, and this requirement was successfully met.

Our strict inclusion criteria limited participation to systemically healthy, nonsmoking young adults with regular interdental hygiene habits. While a healthy baseline oral microbiota minimizes confounding factors and significantly improves internal validity, it inherently limits the direct generalizability of our findings to other population groups.

Assuming that CHX non-selectively reduces oral bacteria, the recolonization dynamics in patients with gingivitis or periodontitis could be different, since the initial state of the oral microbiota in these patients is distinct from that of healthy individuals; therefore, the recovery of the oral microbiota following exposure to CHX may also vary (37). Similarly, older populations or smokers, who have a different oral microenvironment and altered host immune responses, may exhibit different microbial changes following exposure to CHX (38). Therefore, future longitudinal studies are warranted to assess whether the changes in oral microbiota composition observed in our healthy cohort also occur in at-risk groups.

It is essential to consider the exact formulations of the dentifrices used in this study, as they were explicitly selected to reflect typical home oral care conditions. The reference toothpaste, used during the baseline and washout phases, contained amine fluoride, which is not ecologically neutral. While direct evidence regarding its specific effect on the salivary microbiota is limited, studies on dental plaque demonstrate that fluoride formulations actively influence bacterial composition and metabolic pathways (39–42). Given these distinct ecological dynamics, the baseline microbiota in our study represents a state already conditioned by habitual fluoride exposure. Consequently, the observed microbial shifts should be interpreted as the result of sequential exposure (fluoride at baseline, the test formulation during the CHX intervention, and a return to fluoride in the washout phase) rather than the isolated effect of CHX alone. Furthermore, during the intervention phase, participants were exposed to a home-care toothpaste rather than a pure chlorhexidine solution. As detailed in Table 2, this formulation also contained fluoride and cetylpyridinium chloride, a substance known to synergistically enhance the antiseptic effect (43).

A notable limitation of this study is the reliance on 16S rRNA gene sequencing targeting the V3–V4 region. While this approach is a widely established standard for profiling the oral microbiota, it presents inherent methodological constraints compared to whole-genome shotgun sequencing. Specifically, it lacks sufficient taxonomic resolution to accurately identify bacteria at the species level, restricting our findings primarily to broader taxonomic ranks (Supplementary Figure 2). Furthermore, our analysis is based exclusively on relative abundance. Because CHX is an antiseptic known to significantly reduce the overall bacterial load, changes in relative abundance may not accurately reflect the absolute biological expansion or reduction of specific taxa. The absence of total bacterial quantification is a limitation that must be considered.

From a clinical perspective, the use of 0.06% chlorhexidine digluconate toothpaste induced statistically significant changes in the oral microbiota, which became most evident at the end of the 12-week washout phase. While long-term exposure to this low-concentration antiseptic did not increase the prevalence of predicted antimicrobial resistance pathways, taxonomic changes in the oral bacterial community were observed. Clinically, dental professionals should carefully weigh the therapeutic benefits of CHX products against the risk of inducing these delayed compositional shifts. Our results indicate that the appropriateness of CHX for long-term use should be reconsidered, as its ecological impact on the oral microbiota is not neutral even at lower concentrations.

## Data Availability

The datasets presented in this study can be found in online repositories. The names of the repository/repositories and accession number(s) can be found below: https://www.ebi.ac.uk/ena, PRJEB108494 https://doi.org/10.5281/zenodo.19655210, Zenodo.

## Supplementary material

Supplementary Figure 1 Relative abundance of the top 15 bacterial genera across the three study phases. The stacked bar plots represent the salivary microbiota composition at the genus level for each participant (*n* = 11). The study phases are defined as follows: (1) Baseline: samples collected following a 4-week run-in period; (2) Intervention: samples collected after 12 weeks of using 0.06% chlorhexidine digluconate toothpaste; and (3) Washout: samples collected after a subsequent 12-week period using the reference fluoride toothpaste. Only the top 15 genera by mean relative abundance are displayed.

Supplementary Figure 2 Relative abundance of the top 15 bacterial species across the three study phases. The stacked bar plots display the salivary microbiota composition at the species level for each participant (*n* = 11). The study phases are defined as follows: (1) Baseline: samples collected following a 4-week run-in period; (2) Intervention: samples collected after 12 weeks of using 0.06% chlorhexidine digluconate toothpaste; and (3) Washout: samples collected after a subsequent 12-week period using the reference fluoride toothpaste. Only the top 15 species by mean relative abundance are displayed.

Supplementary Figure 3 Statistical validation of metabolic shifts. Error plot of highly significant predicted metabolic pathways (*p*-adj < 0.01) comparing the intervention phase (Phase 2) and the washout phase (Phase 3). The plot displays mean differences with 95% confidence intervals.
