# Supplementary material for: Delayed shifts in salivary microbiota composition following long-term use of 0.06% chlorhexidine toothpaste
Source: Front Oral Health. 2026 Aug 11;7:1887909. doi: 10.3389/froh.2026.1887909 (PMC13503232; doi:10.3389/froh.2026.1887909)
Supplement: Supplementary file 1 [file Table1.docx]

**Supplementary Table 1. Dynamics of predicted peptidoglycan biosynthesis pathways associated with antimicrobial resistance.**

| **PWY-6470:peptidoglycan biosynthesis V** | **Mean** | **Adjusted p-value** |
| --- | --- | --- |
| Phase 1 | 0.213812 | > 0,5 (Phase 1 vs Phase 2) |
| Phase 2 | 0.225174 | <0.002 (Phase 2 vs Phase 3) |
| Phase 3 | 0.161982 | <0.02 (Phase 1 vs Phase 3) |

| **PWY-6471: peptidoglycan biosynthesis IV** | **Mean** | **Adjusted p-value** |
| --- | --- | --- |
| Phase 1 | 0,4099811 | > 0,5 (Phase 1 vs Phase 2) |
| Phase 2 | 0,41532 | <0.002 (Phase 2 vs Phase 3) |
| Phase 3 | 0,351634 | <0.02 (Phase 1 vs Phase 3) |

The phases are defined as follows: ( Phase 1) Baseline: samples collected following a 4-week run-in period using a control toothpaste; ( Phase 2) Intervention: samples collected after 12 weeks of using 0.06% chlorhexidine digluconate toothpaste; and ( Phase 3) Wash-out: samples collected after a subsequent 12-week recovery period using the control toothpaste.
